# UNIPred-Web: a web tool for the integration and visualization of biomolecular networks for protein function prediction

**DOI:** 10.1186/s12859-019-2959-2

**Published:** 2019-08-14

**Authors:** Paolo Perlasca, Marco Frasca, Cheick Tidiane Ba, Marco Notaro, Alessandro Petrini, Elena Casiraghi, Giuliano Grossi, Jessica Gliozzo, Giorgio Valentini, Marco Mesiti

**Affiliations:** 1 Department of Computer Science, Università degli Studi di Milano, Via Celoria 18, Milano, 20133 Italy; 2 Fondazione IRCCS Ca’ Granda - Ospedale Maggiore Policlinico, Università degli Studi di Milano, Via della Commenda 10, Milano, 20122 Italy

**Keywords:** Imbalance-aware protein function prediction, Imbalance-aware protein networks integration, Visualization of protein networks, Web service for protein function and network integration

## Abstract

**Background:**

One of the main issues in the automated protein function prediction (AFP) problem is the integration of multiple networked data sources. The UNIPred algorithm was thereby proposed to efficiently integrate —in a function-specific fashion— the protein networks by taking into account the imbalance that characterizes protein annotations, and to subsequently predict novel hypotheses about unannotated proteins. UNIPred is publicly available as R code, which might result of limited usage for non-expert users. Moreover, its application requires efforts in the acquisition and preparation of the networks to be integrated. Finally, the UNIPred source code does not handle the visualization of the resulting consensus network, whereas suitable views of the network topology are necessary to explore and interpret existing protein relationships.

**Results:**

We address the aforementioned issues by proposing UNIPred-Web, a user-friendly Web tool for the application of the UNIPred algorithm to a variety of biomolecular networks, already supplied by the system, and for the visualization and exploration of protein networks. We support different organisms and different types of networks —e.g., co-expression, shared domains and physical interaction networks. Users are supported in the different phases of the process, ranging from the selection of the networks and the protein function to be predicted, to the navigation of the integrated network. The system also supports the upload of user-defined protein networks. The vertex-centric and the highly interactive approach of UNIPred-Web allow a narrow exploration of specific proteins, and an interactive analysis of large sub-networks with only a few mouse clicks.

**Conclusions:**

UNIPred-Web offers a practical and intuitive (visual) guidance to biologists interested in gaining insights into protein biomolecular functions. UNIPred-Web provides facilities for the integration of networks, and supplies a framework for the imbalance-aware protein network integration of nine organisms, the prediction of thousands of GO protein functions, and a easy-to-use graphical interface for the visual analysis, navigation and interpretation of the integrated networks and of the functional predictions.

## Background

The recent CAFA (Critical Assessment of Functional Annotation) and CAFA2 challenges showed that the integration of multiple data sources plays a key role in the automated function prediction of proteins (AFP) [1–3]. Individual data sources, usually represented as protein networks, often carry complementary information each other, and often a source can be more informative for some specific protein functions and less informative for other functions [4], thus raising the need to integrate protein networks in a function-specific setting —a consensus network produced for each protein function. Moreover, for most protein functions only few annotated proteins are available [5], thus creating a strong imbalance between annotated (positive) and unannotated (negative) proteins. Accordingly, an imbalance-aware integration is also needed. In this context, the UNIPred algorithm (Unbalance-aware Network Integration and Prediction) has been recently proposed [4]: it computes for each input network a function-specific informativeness score, which is then used to build the consensus network. Both the integration and prediction steps in UNIPred take into account the scarcity of positive proteins. The extensive experimental results presented in [4, 6] showed that COSNet and UniPred, the predictive algorithms used by UNIPred-WEB, compared favorably with a large set of state-of-the-art network-based methods, including e.g. GeneMANIA-SW [7], the classical label propagation algorithm [8], MS-kNN, one of the top-ranked methods in the recent CAFA challenge [1], and the eight best methods of the MouseFunc challenge [9].

UNIPred is available as R code, which implements the integration core procedure, whereas the prediction procedure is implemented by the R package COSNet [10]. Both implementations assume that the adjacency matrix and the protein annotations are already preprocessed and transformed into R binary objects. This makes not immediate the usage of UNIPred for a generic user, which is required to retrieve the input information (the protein pairwise similarities and the function to protein associations) and to transform it into suitable R matrices, in addition to processing and supplying to COSNet the output of the integration step. Furthermore, the integrated network might contain thousands of nodes and edges, and the matrix format returned by the available R code is far from being of immediate interpretation for the user.

The UNIPred-Web tool is proposed to specifically overcome these limitations. A collection of around two thousand heterogeneous networks has been retrieved from the literature and prepared for the integration —networks cover nine prokaryotic and eukaryotic organisms. The system also allows the upload of user-defined networks. A graphical interface guides the user during the selection of the organism, the GO protein function, the input networks, and eventually the proteins to be predicted (see “Experimental setting interface” section). The experiment is then submitted to a scheduler, which manages the requests of different users and allocates the required resources. An email is sent to the user when the integration process is completed, and the user can then visualize and explore the resulting network. The visualization starts from a target protein selected by the user, and it allows to interactively personalize the resulting subgraph —the user can easily expand or reduce the graph size, move nodes, see information associated with nodes and edges, and apply different visualization options (see “Visual analysis and exploration of the integrated networks” section).

We added an Appendix to include some UNIPred-Web tool usage scenarios to integrate biological networks, explore the subnetwork centered on a specific target protein, load user-defined networks, visualize the predictions with respect to a GO term, and enlarge the visualization of the subnetwork in order to conduct further analyses.

## Implementation

In this section we firstly provide a description of the input networks which are made available by UNIPred-Web. Note that users can either use, integrate, and explore the provided networks, or can provide their own networks. Secondly, we describe the algorithmic engine behind UNIPredWeb. Specifically, we discuss UNIPred [4] for networks integration, and COSNet [6, 11] for protein function predictions.

### Networks and organisms

Input networks in UNIPred-Web have been retrieved from the literature, following the schema proposed in [7] and adopted by the GeneMANIA server [12], where protein networks are grouped by type, including *co-expression* (GEO [13]), *co-localization* (LocSigDB [14]), *genetic interactions and pathways* (NCI-Nature Pathway Interaction Database [15]), *physical interactions* (BioGRID [16], MINT [17], and IntAct [18]), *protein domain profiles* [19, 20]. Moreover, to obtain more accurate predictions, UNIPred-Web also includes networks from the STRING v10 database [21], which supplies networks (one for each organism) already merging several sources of information into the pairwise protein connections (e.g. sequence homology, textmining, and co-expression). Ensemble protein identifiers are adopted to represent proteins with frequently used aliases (when available).

Available networks belong to nine different organisms: *Escherichia coli* (NCBI taxonomy id 562), *Arabidopsis thaliana* (3702), *Saccharomyces cerevisiae* (4932), *Caenorhabditis elegans* (6239), *Drosophila melanogaster* (7227), *Danio rerio* (7955), *Homo sapiens* (9606), *Mus musculus*, (10090), *Rattus norvegicus* (10116). Functional annotations are downloaded from the GO repository, by considering the latest UniProt GOA release for every organism [22]. Only experimentally validated associations are retained.

### The integration engine

For a given organism, the network integration problem consists in merging every selected network *k*, represented through a weighted undirected graph *G*^(*k*)^=〈*V*,***W***^(*k*)^〉 on the proteins/vertices *V* (or a subset of it) and connections ***W***^(*k*)^, into a consensus network *G*=〈*V*,***W***〉 integrating all available networks. Given a GO function *d*, every protein *i*∈*V* holds a label *y*_*d*_(*i*)∈{0, 1} denoting that protein *i* is currently associated with *d* (label 1, positive protein) or not (label 0, negative protein). Integrating networks specifically for a GO term *d* requires associating every network *G*^(*k*)^ with a coefficient $r_{d}^{(k)}$ related to its informativeness for *d*, and then linearly combining all networks using the computed coefficients.

UNIPred allows the construction of a dedicated composite network for each GO term, and is able to capture the predictive capability of single networks in classifying positive proteins, by giving more weight to the networks which carry most information. More precisely this method operates a network projection onto the plane so that each protein *i*∈*V* is associated with a labeled bi-dimensional point $P_{i}^{(k)}$, embedding the local imbalance in the corresponding node position. The coordinates $P_{i}^{(k)}\equiv \left (P_{i,1}^{(k)};\ P_{i,2}^{(k)}\right)$ are computed as:

$$P_{i,1}^{(k)} =\sum_{j\in V} {W_{ij}^{(k)}\cdot y_{d(j)}} ~~~~~~~~~~ P_{i,2}^{(k)} =\sum_{j\in {V}} {W_{ij}^{(k)}\cdot (1 - y_{d}(j))}$$

In other words, $P_{i,1}^{(k)}$ is the weighted sum of positive neighbors, while $P_{i,2}^{(k)}$ is the weighted sum of negative neighbors. The position of each point in the plane thereby reflects the topology of the connections towards neighboring positive and negative nodes. The algorithm then learns the straight line which best separates positive and negative points, in the sense we describe below. Since every point *i*∈*V* already has a label *y*_*i*_, each line separating positive and negative points is associated with the number ${TP}_{d}^{(k)}$ of positive points correctly classified (true positives) for the term *d*, the number ${FN}_{d}^{(k)}$ of positive points wrongly classified (false negatives), and the number ${FP}_{d}^{(k)}$ of negative points wrongly classified (false positives). The optimal line is the one maximizing the *F*–measure:

$$F_{d}^{(k)} = \frac{2{TP}_{d}^{(k)}}{2{TP}_{d}^{(k)}+ {FP}_{d}^{(k)} + {FN}_{d}^{(k)}}\;. $$

The value $\bar F_{d}^{(k)}$ corresponding to the optimal line is then considered as relevance $r_{d}^{(k)}$ for the input network *G*^(*k*)^. The method is imbalance-aware since the *F*–measure by definition penalizes more heavily the misclassification of positive instances, with respect to the penalty for misclassifying negatives. Moreover, maximizing $F_{d}^{(k)}$ moves the known labeling ***y***_***d***_=(*y*_*d*(1)_,…,*y*_*d*(|*V*|)_) towards a minimum of the energy of the underlying Hopfield network — allowing the model to better fit the input data (see [4]). The overall execution time obviously depends on the number and the size of the networks to be integrated; to speed-up the computation, the time consuming procedures are implemented in **C** language.

### The prediction engine

Once the consensus network has been obtained, solving the prediction problem for the selected GO functional term *d* and for a user-selected set of proteins *U*⊂*V* consist in: 1) computing a score function $\phi : U \longrightarrow \mathbb {R}$, which ranks proteins *U* so as to assign higher scores to proteins more likely to be associated with *d*; 2) to determine a bipartition (*U*^+^,*U*^−^) of queried proteins respectively into the sets of proteins being putatively annotated or not with the function *d*.

If the user has not specified a list of proteins to be predicted, the algorithm ends and the user can proceed with the visualization tool; otherwise, the prediction algorithm is invoked, which will provide both the protein rankings (according to function *ϕ*) and the classification of queried proteins— bipartition (*U*^+^,*U*^−^)— (see “Visual analysis and exploration of the integrated networks” section for a description of the visualization results). Even to predict the selected proteins *U* (or all available proteins in the case the user chose this option) UNIPred-Web adopts an imbalance-aware classifier: the *COSNet* algorithm, a state-of-the-art method specifically designed to predict protein functions by coping with the label imbalance affecting GO terms and having performance competitive with the state-of-the-art methodologies proposed for AFP [4, 6, 11]. An extension of COSNet, originally proposed as a binary classifier, is adopted to infer also the protein ranking *ϕ* [23, 24]. The function *ϕ* corresponds to the internal neuron energy at equilibrium, normalized in the range [−1,1]: the higher the score, the higher the likelihood that the protein possesses the given GO function. Intermediate scores (nearby 0) correspond to more uncertain predictions. We used the **R** package of COSNet [10] that efficiently implements in C language the Hopfield network dynamics and parameters learning procedure.

## Results

In this section we describe the UNIPred-Web facilities for the specification of network integration, for the visualization and exploration of the integrated network. The different options that can be exploited by the user for the personalization of the visualization are discussed along with an usage example. Finally, we compare our system with the state of the art and outline its peculiarities.

### Experimental setting interface

**Fig. 1:**
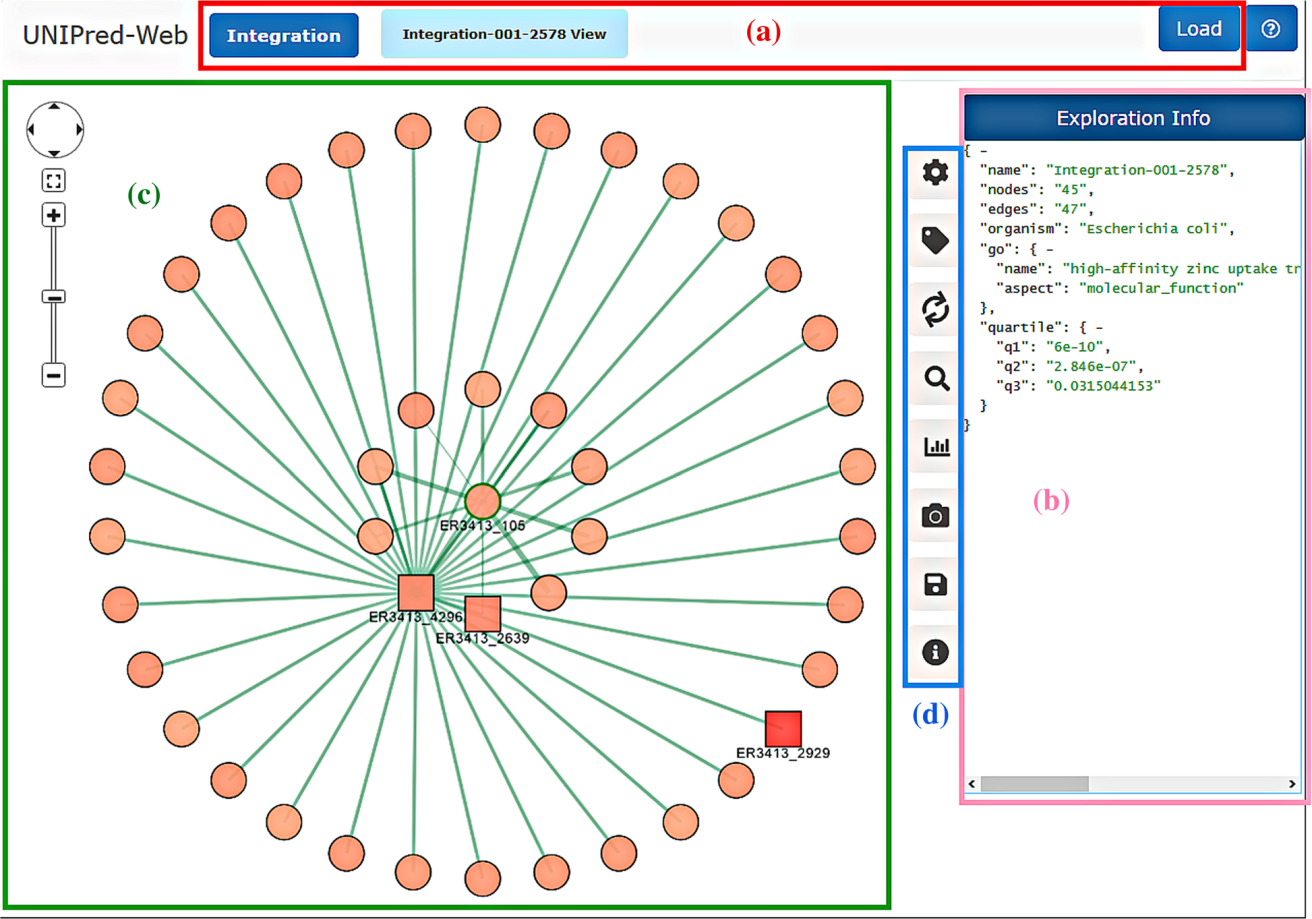
Overall organization of the UNIPred-Web application. The area (**a**) allows the specification of the networks to be integrated and the target protein from which the integrated network exploration should be started. The area (**b**) reports details of the integrated network. The area (**c**) is the canvas where the graph is drawn and can be manipulated. The area (**d**) reports the operations that can be applied on the integrated network

**Fig. 2:**
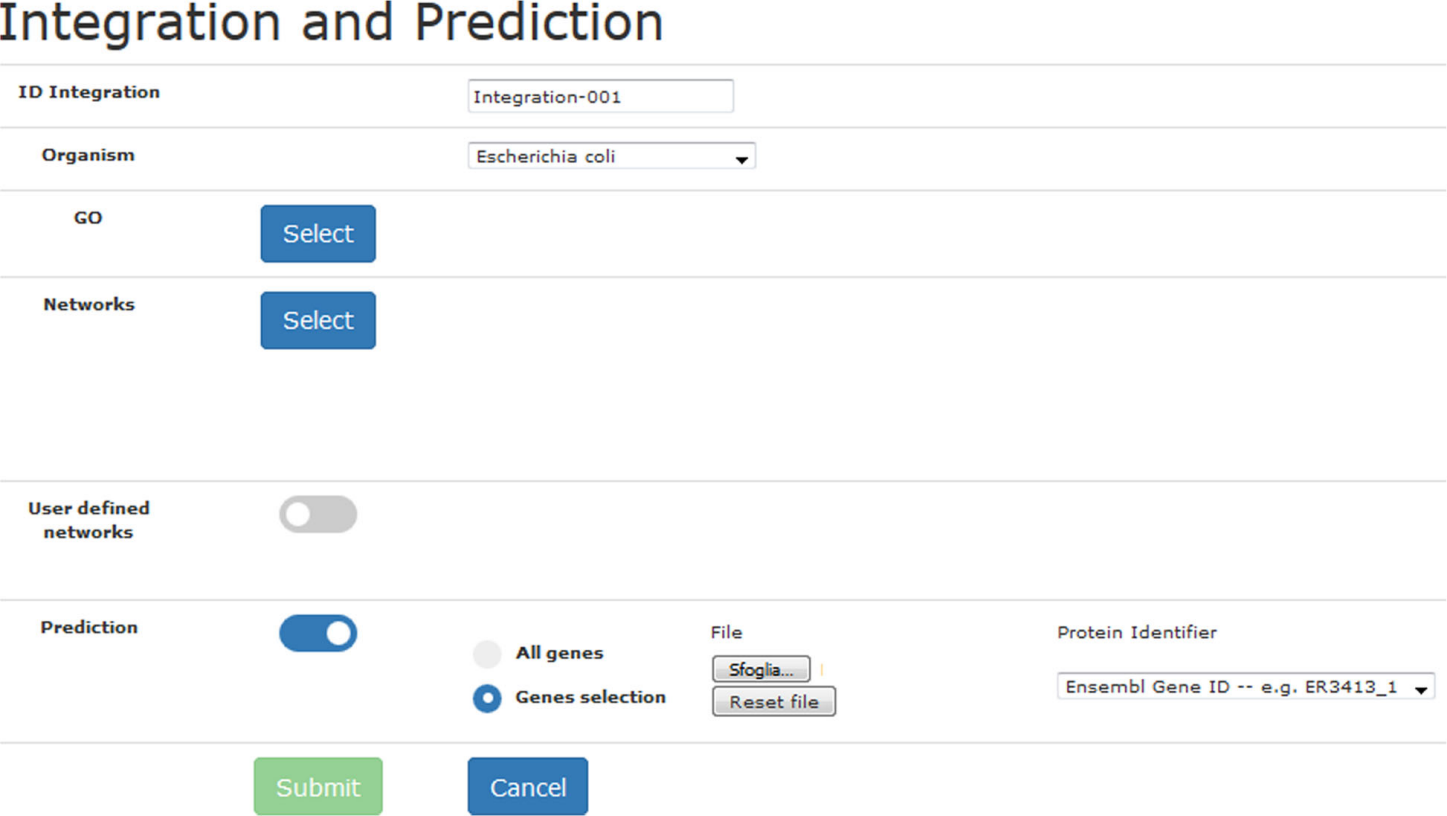
Form for the specification of the networks integration and prediction

Figure 1 shows the starting panel of UNIPred-Web which is available at http://unipred.di.unimi.it. In the top-left corner (area *a*) there is the “integration” button that allows the specification of the integration and prediction activities, as shown in Fig. 2.

**Fig. 3:**
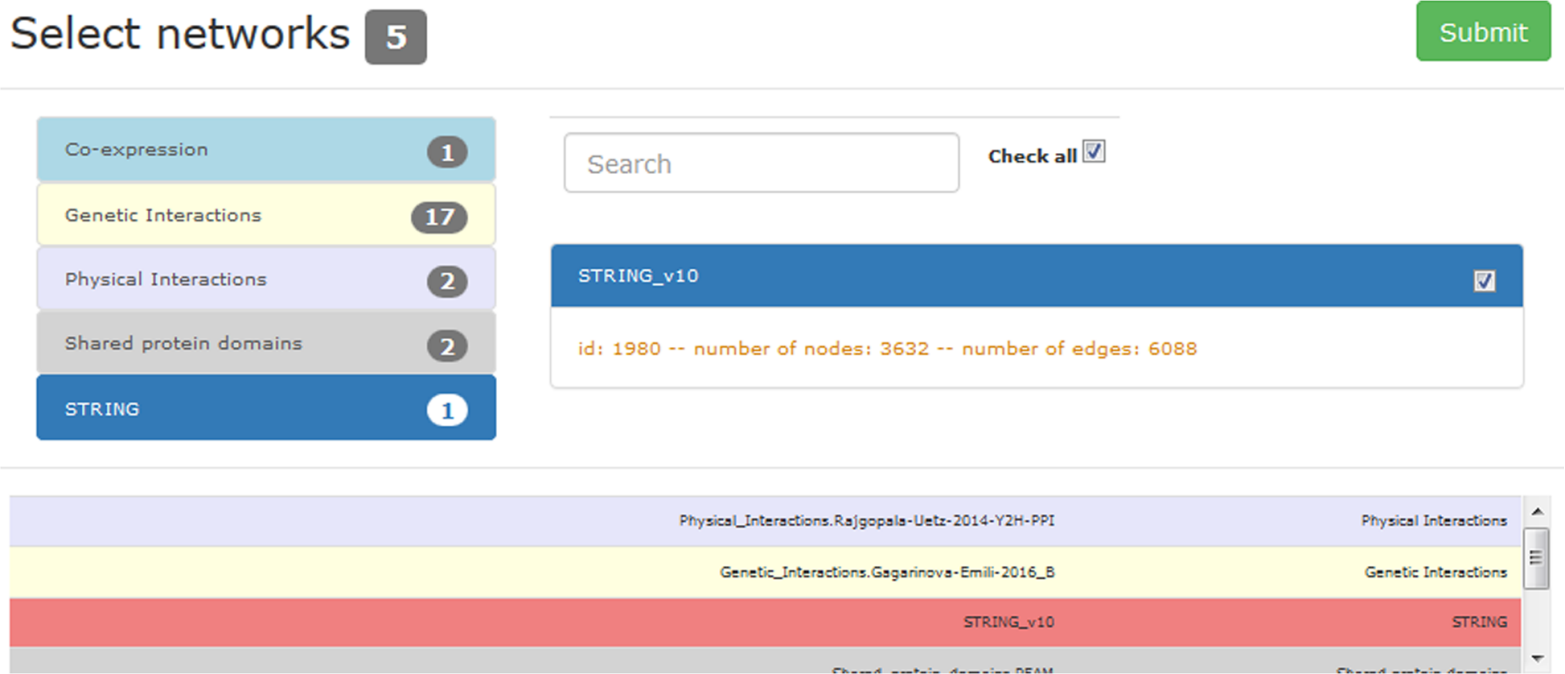
Web interface for the selection of networks

A system-generated name for the current experiment is proposed, that the user can personalize (this is the reference to be exploited in the visual analysis). Once the organism and the GO term of interest are selected, the interface allows the specification of the networks to be integrated: a default set of networks has been pre-selected for each organism, and radio buttons are available to select/remove individual networks or to select/remove all the networks of a specific type (Fig. 3). The selection is based on the source type (e.g. co-expression, co-localization, genetic interaction) or on the network name (by means of the text search box in the top of the form). For each network, the name, and the number of nodes and edges are reported.

Users can also upload their own network by activating the toggle switch “User defined network” (Fig. 2). The network must be supplied in the triplet tab-delimited text format (the required format is explained in the help tab on the top-right of the interface in Fig. 1 and an example is reported in Fig. 15 the Appendix.). Through another toggle switch, the user can request the prediction of the association of proteins with the GO term selected: the prediction can involve all the proteins, or alternatively a subset of proteins specified by the user in a newline-separated textual file. UNIPred predictions are both binary (associated/non-associated) and real-valued (a real score such that the higher the value, the more likely is the association between the protein and the GO term). The user-defined network and functional prediction facilities are optional. Finally, the system requires an email address to send a notification at the end of the execution. The computation is run in batch mode, allowing the user to plan a novel integration, or to navigate the output of previous experiments.

### Visual analysis and exploration of the integrated networks

**Fig. 4:**
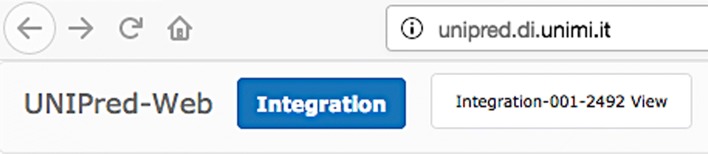
Accessing to integration results

When the process is completed, the system allows to access the result through a dedicated button in the navigation bar (button *Integration 001-2492 View* in the example in Fig. 4). The button is shown automatically when the computation is done on the fly, or after loading the experiment by specifying the code reported in the notification e-mail (“load” button, top-right Fig. 1).

**Fig. 5:**
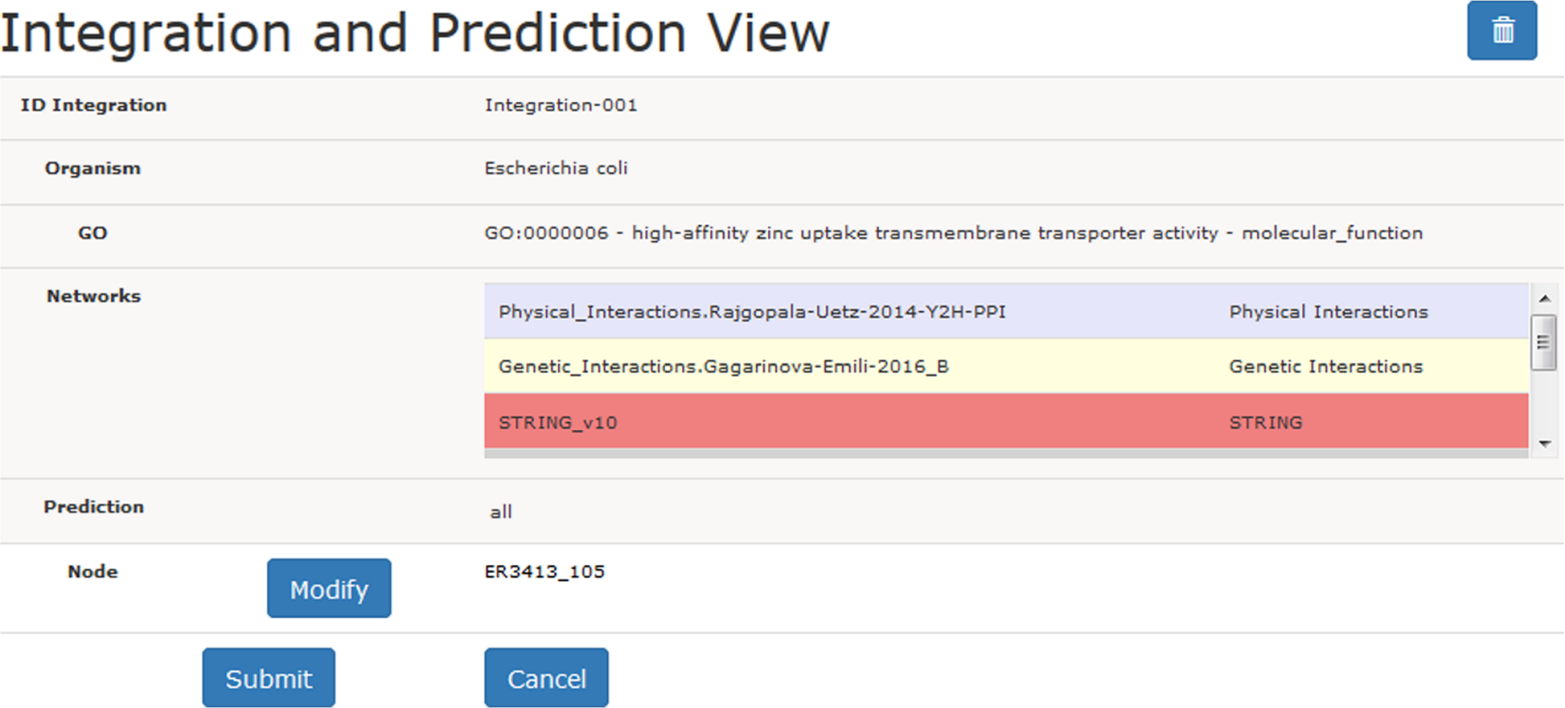
Web interface for starting the navigation of the integrated network

In the form displayed (Fig. 5), the user specifies the target protein from which the exploration should start. The subgraph of nodes connected to the target node is then visualized. Showing a reduced portion of the integrated network allows a better visualization of the local characteristics of the network around the target protein.

**Fig. 6:**
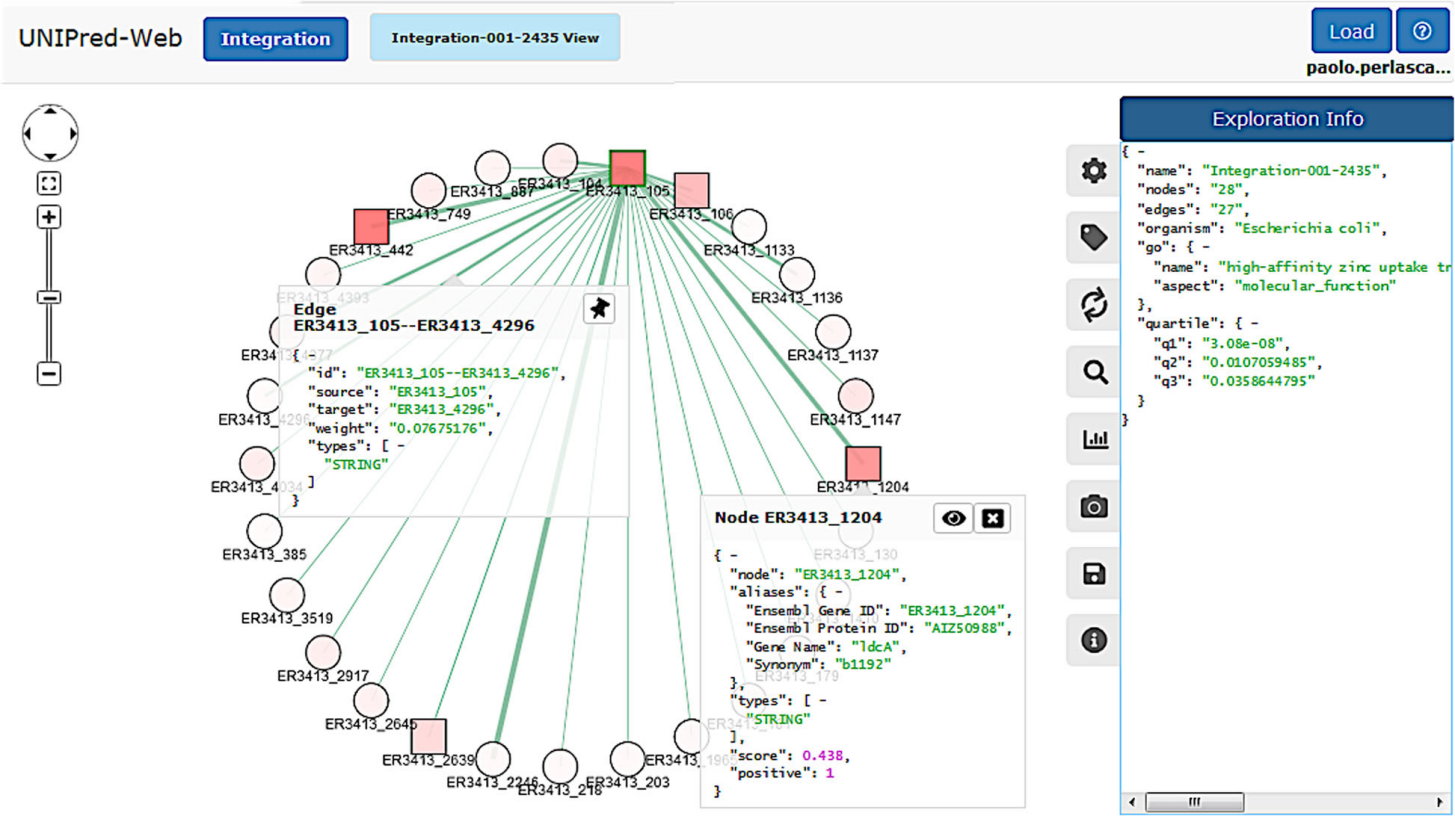
Vertex-centric exploration of the integrated network and information provided for each node and each edge

Figure 6 shows an example of rendering of an integrated network that is centered on the *E.coli* protein ER3413_105.

Depending on the selection of the prediction option, the rendering of the resulting graph changes as follows:

- *No prediction*. All nodes are drawn as white circle.
- *Prediction all*. All nodes are colored and the color graduation reflects the prediction score assigned to the protein. Moreover, nodes can have a different shape: a square is used for annotated protein that are instances of the GO class, whereas a circle is used for the other proteins.
- *Prediction selection*. The nodes for which a prediction is requested are represented through the colored square or circle nodes (as we have done for the Prediction all case). All the others nodes are represented as white circles.

To get information about a protein/edge shown in the canvas, the user just needs to click on it. In Fig. 6 the system shows for the protein ER3413_1204, some main alias identifiers, the type of node and, in case of prediction, both the binary and real-valued predictions. For the edge connecting the proteins ER3413_105 and ER3413_4296, the system reports the target nodes, its weight, and the network sources in which it is actually present. At this stage, to improve the visualization, the user is allowed to drag each vertex within the canvas to obtain a personalized view.

### Interacting view

**Fig. 7:**
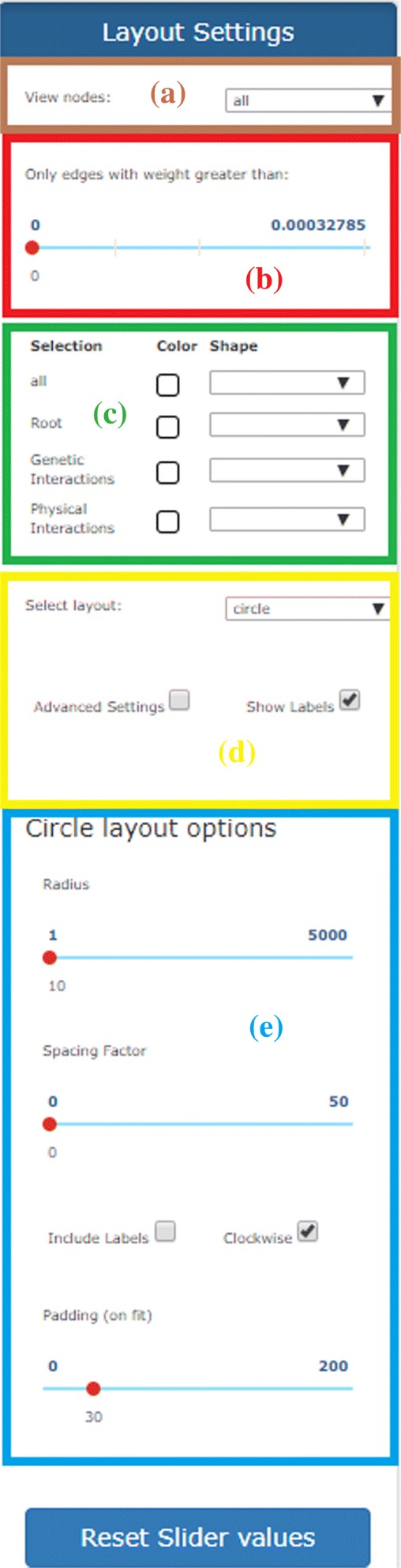
Panel for the personalization of the network visualization. (**a**) Panel for selecting nodes to be shown in the canvas; (**b**) panel for removing edges based on their weights; (**c**) panel for choosing the colors and shapes of nodes/edges. (**d**) panel for layout selection; (**e**) panel for specifying options to improve the chosen visualization
- *Removing edges relying on their weights* (area *b* in Fig. 7). By using the bar, only the edges whose weight is above a given threshold are maintained in the canvas. This feature is quite useful for keeping in the canvas only the edges with higher connectivity relevance.
- *Colors and shapes of nodes/edges* (area *c* in Fig. 7). A set of buttons and check boxes are provided for controlling the color and/or the shape of the nodes in the canvas according to their source type. In this way, the user can highlight the contribution given by individual sources to every connection in the integrated network —for instance, the user can select the subset of nodes/connections present just in co-expression networks, or present in co-expression and/or physical interactions networks.
- *Selection of the layout* (area *d* in Fig. 7). The web tool is equipped with different visualization options (layouts) for making the analysis of the generated network more user-friendly. The most interesting are the *cose*, *grid*, *concentric*, *circle* and *breadthfirst* layouts (discussed below). Once selected the layout, some options can be specified for improving the current visualization (area *e* in Fig. 7). We have selected a set of basic parameters that can be used by non-experts users. By clicking on the advanced settings checkbox, these basic parameters can be customized for improving the visualization. Such a feature is specifically designed to appropriately deal with networks of big size. As an example, in Fig. 8a is shown a network with the default settings, whereas, in Fig. 8b we show the result of the manual adaptation obtained by applying the visualization options. As the reader can see, the black cloud of nodes is separated in three well shaped clusters of nodes.

By clicking on the settings button (first button in the area (*d*) of Fig. 1), the panel in Fig. 7 is shown. This panel allows the personalization of network visualization from different perspectives:

- *Selection of visible nodes* (area *a* in Fig. 7). By using this drop-down menu it is possible to view in the canvas the entire set of nodes or limiting the view according to the specific node type.

**Fig. 8:**
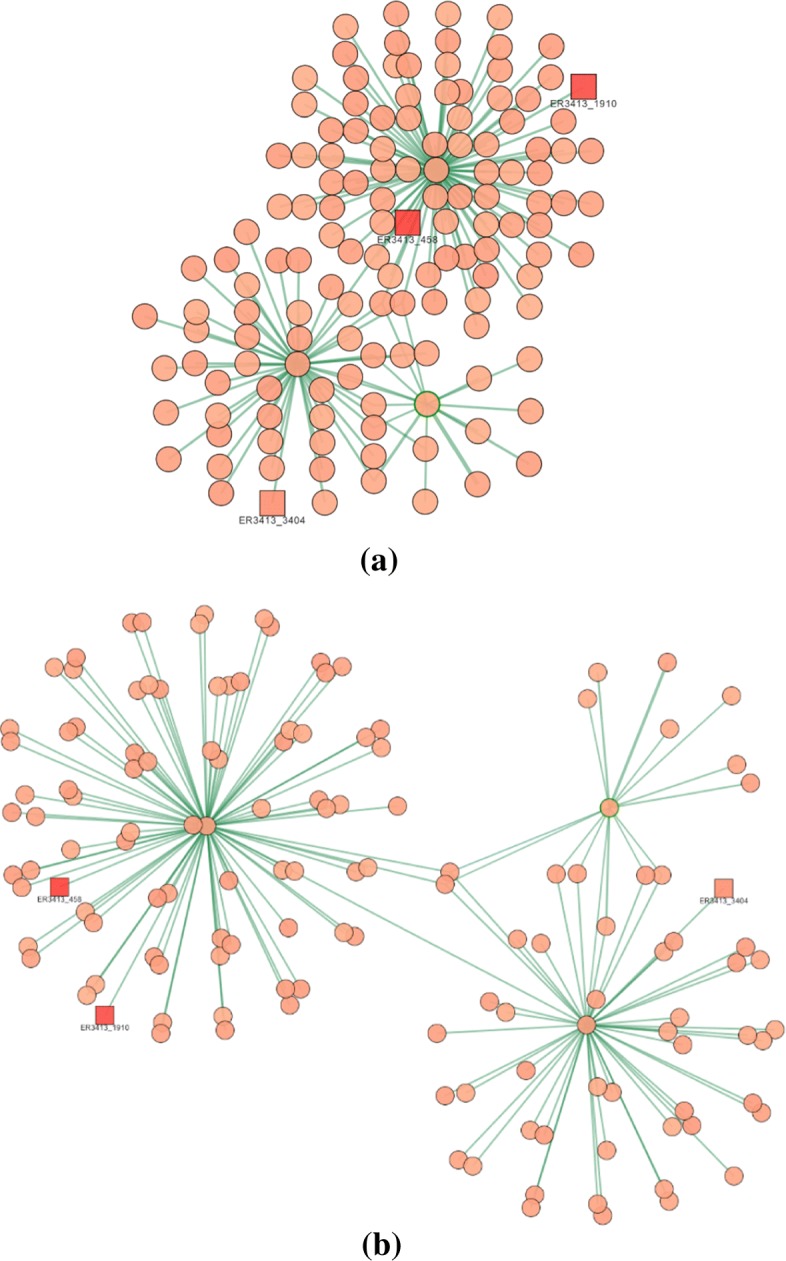
Cose layout. **a** default visualization; **b** advanced settings option selected

**Fig. 9:**
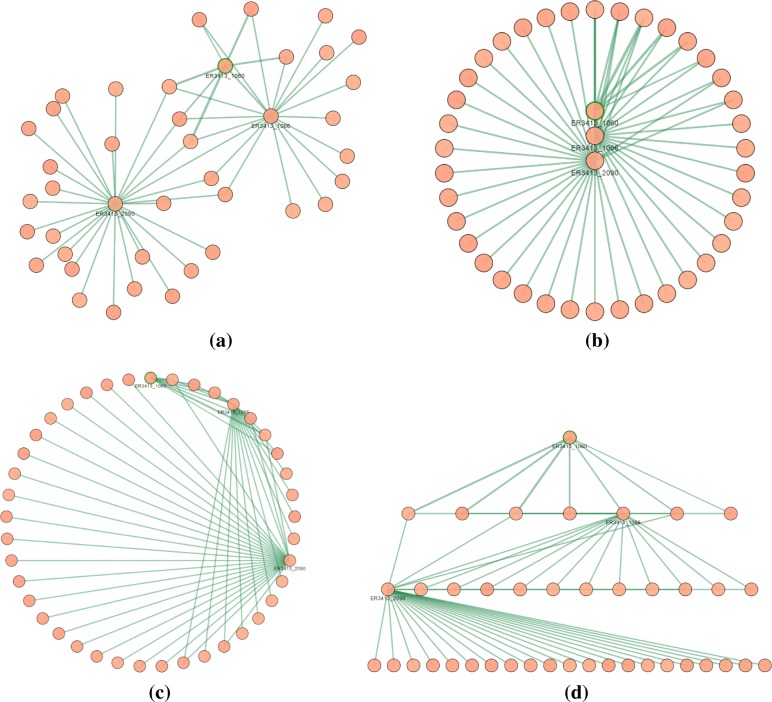
Layout visualization options applied to the same network. **a** Cose. **b** Concentric. **c** Circle. **d** Breadthfirst

In our work we have exploited the layouts made available by the Cytoscape.js library that, in some cases, have been enhanced for working with our weighted networks. Figure 9 shows the application of a selection of different layouts to the same network. Each layout depends on several options whose values determine the actual rendering of the network; for each layout there is a basic and an advanced setting group of options. In general, the advanced setting version increases the effects of each option but sometimes it can change how nodes are ordered into the graph rendering: as an example, the graph in Fig. 8b is obtained from the graph in Fig. 8a by increasing the *node repulsion* option. The *cose* [25] visualization option leverages a physics simulation based on the traditional force-directed layout algorithm with extensions handling multi-level nesting. With the *grid* visualization option, the proteins in the subnetwork are placed in a grid and their connections are shown in the canvas. This rendering offers to the user the possibility to visualize groups of proteins tending to form highly connected components. With the *concentric* visualization option, the target protein is positioned at the center of the canvas and vertices at distance one, two or three are drawn in different concentric circles, as shown in Fig. 9b. This rendering allows the user to better understand the connectivity of the target with its neighborhood and how the functional annotations are propagated from the annotated proteins to the others. In the default mode, the level of a node corresponds to the degree of the node. The nodes with the highest degree are positioned towards the center, while those with the lowest degree are inserted towards the outside. If two nodes have the same degree, they are inserted in the same level. However, it does not guarantee that the root node is inserted in the middle of the view. In advanced mode, the nodes are positioned according to the distance from the node indicated as "root" of the experiment. Nodes at the same distance from the root are positioned on the same level. With the *circle* visualization option, all vertices are posed in a circle: vertices with a higher in-out-edge-degree are positioned closer in the circle. In the default mode, nodes are reordered according to the degree while in the advanced one, the sorting function changes: the nodes are positioned in ascending order of weight. This visualization, as shown in Fig. 9c, allows to better appreciate the nodes for which there is a high interconnection strength from those whose connections are minimal. This feature might help to graphically detect *hub proteins*, i.e. those possessing higher centrality indexes, such as node degree, betweenness, and local clustering coefficient. For instance, node degree has been shown being a proxy for gene multifunctionality [26]. Finally, the *breadthfirst* visualization option puts nodes in a hierarchy, based on a breadth-first traversal of the graph, as shown in Fig. 9d.

### Node-specific options

The graphical view can be further personalized by operating on single nodes. Left-clicking on a node allows to drag the node in a different position in the canvas; right-clicking on a node displays the following choices:

- *Pin the tooltip*: the tooltip is kept in the canvas.
- *Close tooltip*: the corresponding tooltip is closed.
- *Center view on this node*: the current network is redrawn in the canvas by positioning the current node at the center of the canvas.
- *Show/hide this label*: it allows to hide or show the label associated with the current node.
- *Lock/unlock this node*: it allows to fix the position of the current node (eventual modifications of the layout do not affect the current node position).
- *One step from here*: it allows to include in the visualization the nodes that are a step-forward from the current node. Whenever no nodes can be added, an alert is given to the user. This facility is particularly useful for the exploration of the subnetwork, since only nodes one-edge far from the target node are shown by default (to limit the number of nodes to be displayed); this option allows thereby the user to explore other parts of the network not shown in the default visualization.

### Visualization facilities

Table 1 reports the available facilities on the right side of the canvas. Moreover, further facilities have been developed for searching the integrated network and for the management of predictions. Specifically:

- *Searching on the integrated network.* Since the number of nodes and edges in the canvas can be high, the system provides users with a search function for both nodes and edges. In the first case it is possible to specify part of the name of a node to filter the data, while in the second case it is also possible to filter the edges on the basis of their weight, as shown in Fig. 11. In both cases, clicking on a node/edge, the system highlights the position of the selected item in the canvas by opening the correspondingtooltip.

**Table 1:** Operations to be applied on the integrated network

Name	Symbol	Options	Description
Labels		None	The labels on the nodes can be shown or hidden.
Save			The integrated network currently displayed in the canvas can be download in different compressed formats (csv, json).
Search			Node and edge search relying on node ids. In case of edge search, it is possible to specify one of the ids of its extremes. When the node/edge is identified, the visualization is focused on it, a window is opened containing details of the selected element.
Settings		None	It allows to open/close the panel on the right hand side of the canvas with the visualization options.
Save image		None	The network currently shown in the canvas is saved in PNG format.
Refresh		None	Layout refresh (the position of the nodes is computed again).
Prediction			A table is shown containing the prediction of the edges. Two option: *iCurrent visualization*: only the prediction values of the nodes contained in the canvas are reported; *iiIntegrated network*: the prediction values of the entire integrated network are reported.
Info		None	A window with the information related to the current page is visualized.
- *Visualizing the prediction output.* For what concerns the predictions, UNIPred adopts two different type of outputs according to the user selection: a) subset of vertices specified in the uploaded text file; b) all available vertices. In both cases the system allows to display and save the prediction results both for the subnetwork present in the canvas and for the whole integrated network. Figure 12 shows an example of displaying the results of the prediction for the running example.

### An usage example

**Fig. 10:**
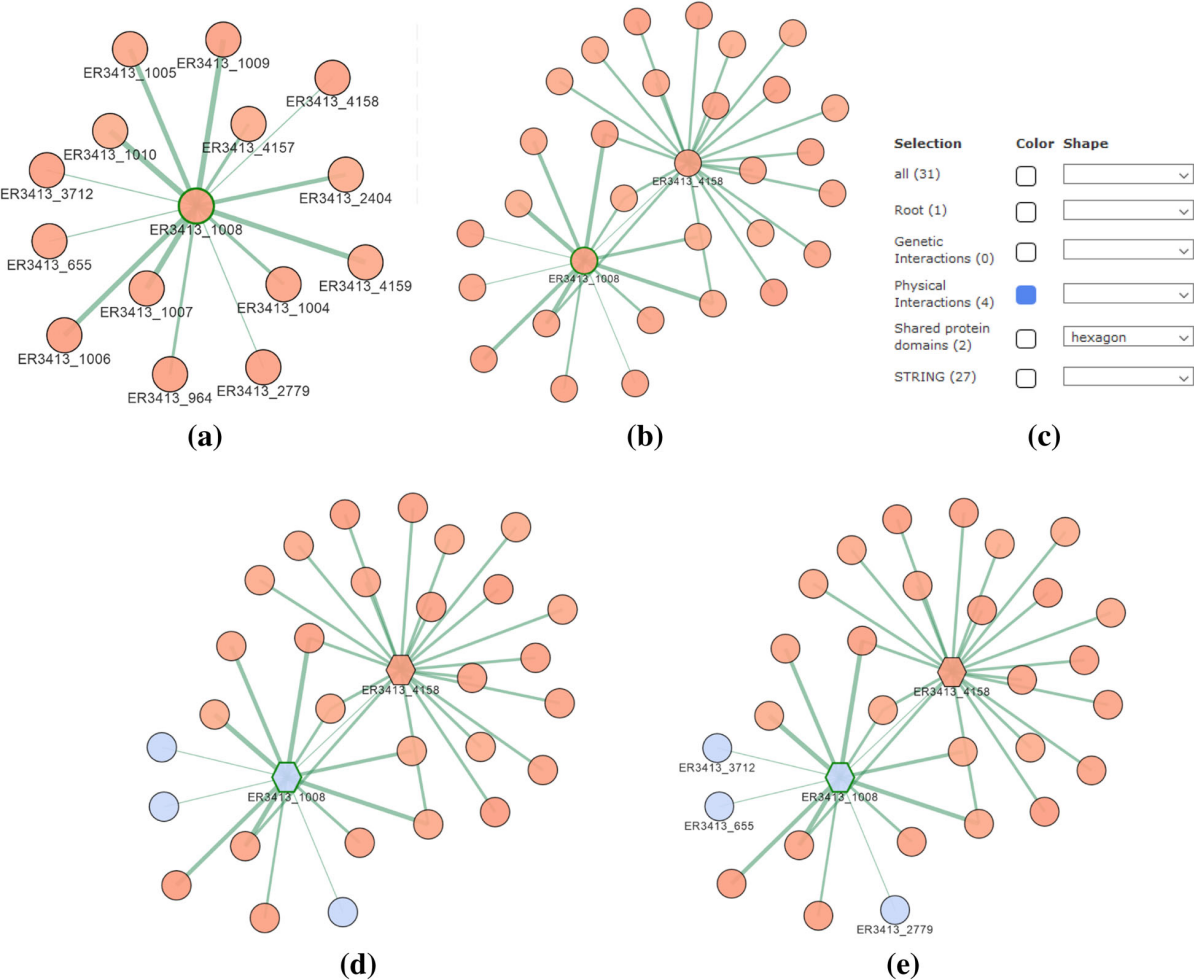
By clicking on the node ER3413_4158 of the network in (**a**), its neighborhood vertices are reported in (**b**) where all the labels except those belonging to particularly interesting nodes are hidden. In (**d**) the modifications described in (**c**) are applied. As a result, the nodes are highlighted by changing color and shape, according to a specific biological functions. In (**e**) all the *physical interaction domain* nodes labels are displayed

Suppose that the networks reported in Fig. 3 have been integrated with the prediction all option and that by means of the interface in Fig. 5 the user has started the navigation of the integrated network from the protein ER3413_1008. The subgraph in Fig. 10(a) is shown that is centered in the node ER3413_1008 and reports the proteins in its neighborhood (those that are directly related with it). By exploring the properties of these proteins, the user can decide to expand the neighborhood of node ER3413_4158 and the expanded network in Fig. 10b is shown. In order to better analyze the structure of the sub graph, in this figure all node labels with the except for nodes ER3413_1008 and ER3413_4158 are hidden. By appropriately clicking the check boxes in Fig. 10c, the user can customize the visualization by changing the shape of the nodes also originated from a *shared protein domain* network (representing them through an hexagon) and by changing the color of the nodes also presenting in a *physical interaction domain* network. Finally, by acting on the tooltips of some nodes, he can display all the *physical interaction domain* nodes labels (Fig. 10e). Further usage examples are available in the Appendix.

**Fig. 11:**
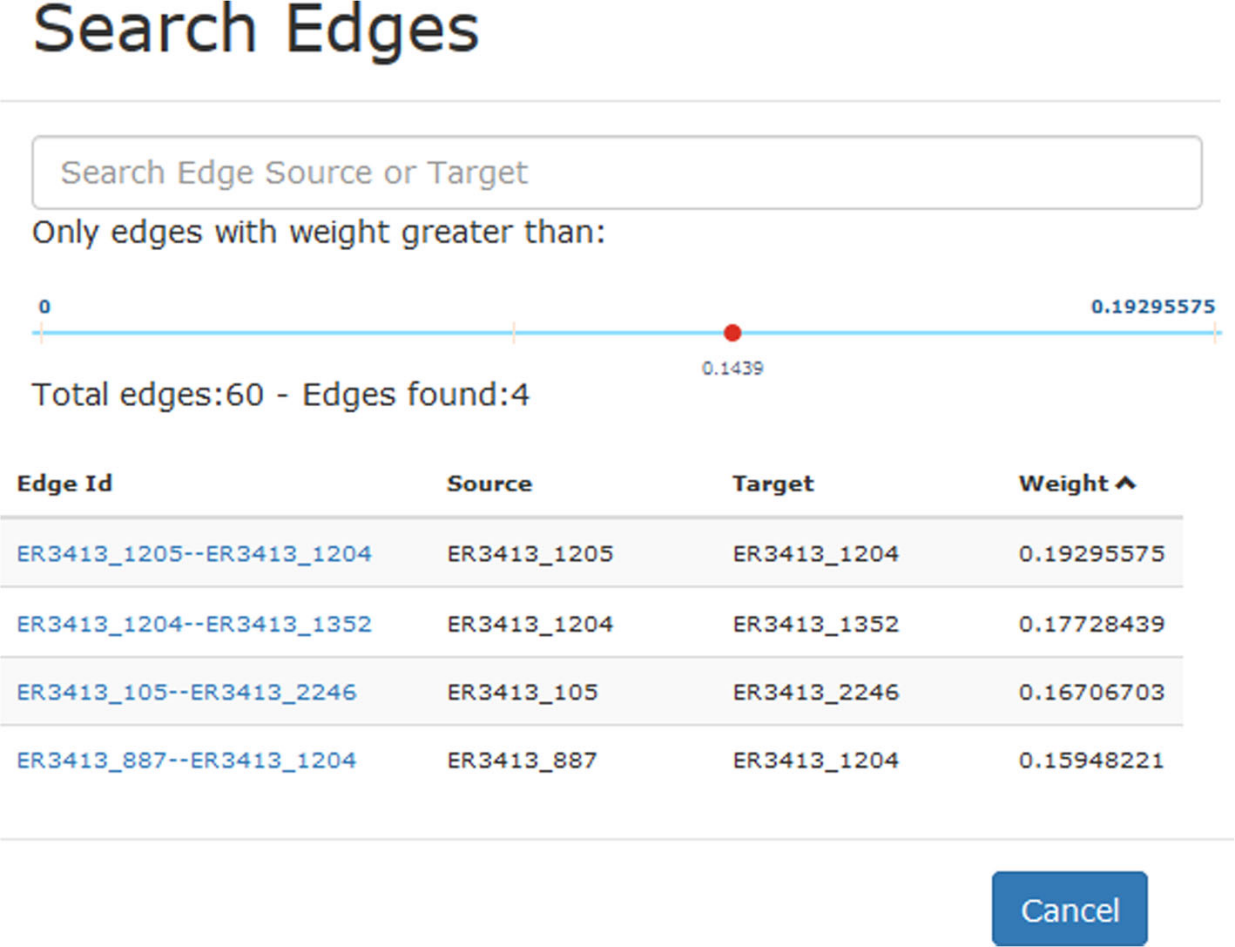
Web interface for searching edges

**Fig. 12:**
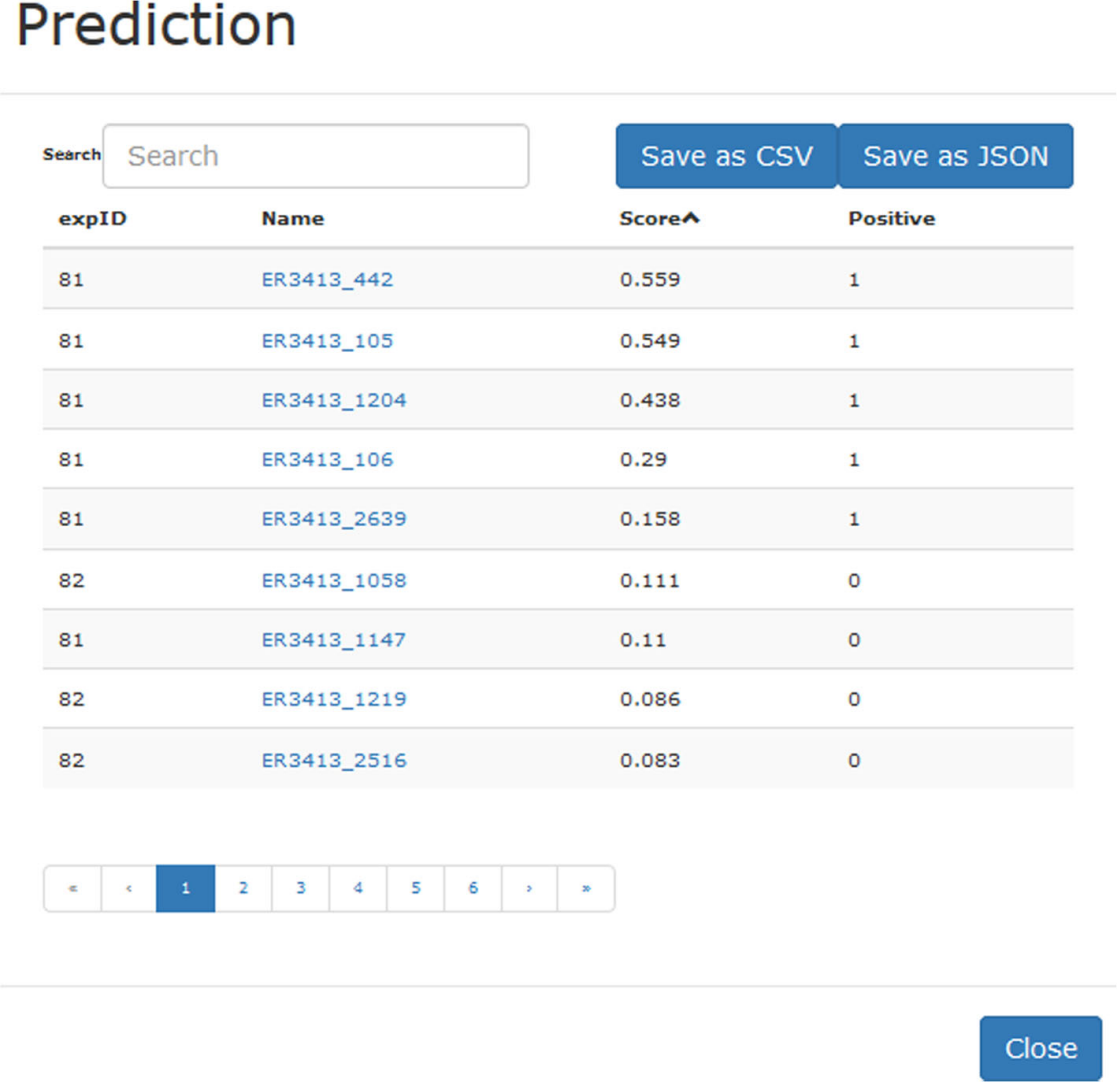
Exploration of the prediction results

### Protein function prediction approaches and network integration web tools

Different web-tools for protein function prediction supplying also network integration are available, including the CombFunc [27], INGA [28], N-Browse [29], SIFTER [30], MouseNet v2 [31], the IMP tool [32], and the GeneMANIA server [33]. CombFunc and INGA combine sequence similarity, protein domain information, protein–protein interaction, and gene expression data to assess the protein function, but do not provide a graphical view of the underlying protein network. N-Browse provides a graphical user interface (GUI), supporting interaction in the visualization of nodes and edges, and allowing the user to select the networks involved in the analysis; however, solely three organisms are supported, and N-Browse runs as a Java web service, which might be not immediate for a generic user. SIFTER is a sequence-based web interface exploring a protein family’s phylogenetic tree as a statistical graphical model of function evolution. The search is limited to one protein at a time, or must include the whole proteome, and the user cannot specify a subset of query proteins. MouseNet v2 extends MouseNET [34], a previous prediction server for laboratory mouse, by including new microarray data derived from diverse biological contexts and embedding other eight model vertebrates to exploit the orthology-based projection of their genes on MouseNet. However the search is limited to one organism. The IMP system provides an easy-to-use interface to query one or more proteins at the same time, even from different organism, by exploiting gene homology information. SIFTER, MouseNet and IMP hide the data integration phase to the user, which consequently cannot evaluate the impact of specific connection types on the final integrated network. Moreover, they do not provide the user with the possibility to interact with the resulting integrated protein network.

Finally, the GeneMANIA prediction server allows the user to specify customized queries, to interact in the visualization process, and provides a graphical view of the obtained consensus network. Nevertheless, it assigns weights using a Gaussian random field methodology, in which the label-imbalance characterizing the GO terms is not handled. The work presented in this paper deeply extend the characteristics of the system proposed in [35]. Specifically more functionalities have been proposed for the rendering of the networks and for loading a user-defined protein network.

## Conclusions

In this paper we presented the features of a Web application for the integration, visualization, analysis and navigation of biological networks. The application has been realized by integrating different technologies: Php and Javascript with AngularJS, Node.js and Cytoscape.js for the client-side and server-side management and visualization of networks. A MySQL database is used to keep track of the curated networks and of the results of the integrated corresponding views. At the current stage the Web application is fully supported by the last version of Firefox and Chrome in different operating systems (Windows 10, Mac OS Yosemite, Ubuntu 18 LTS) and partially supported by Edge. By adopting a vertex-centric rendering of a subnetwork, the system offers different customized visualizations that can be exploited for identifying useful patterns in the analyzed network. The vertex-centric visualization allows the user to focus on the interactome of specific proteins, but at the same time the user can also easily extend in an interactive way the subnetwork to be visualized by simply clicking on the nodes of the network itself. UNIPred-Web is able to compute and visualize protein function predictions for a large set of model organisms, through the integration of different types of interaction networks available from the server or supplied by the user. As future work we are planning to introduce machine learning algorithms for suggesting to the user the best visualization by considering user feedback in the proposed visualizations. Moreover, we plan to model network visualization at different resolution levels for reducing the amount of vertices to be included in the current canvas and allowing a hierarchical visualization of big biological networks. Note that all these demanding applications can be deployed in virtualized environments combined with hardware accelerators [36]. Finally, more sophisticated approaches for the integration of networks will be explored following the design approach adopted in [37].

## Appendix

## Usage scenarios

In this Appendix we introduce some scenarios for better presenting the capabilities of UNIPred-Web. The detailed presentation can be useful for non-expert users that wish to conduct simple visual analysis of the integrated networks. Further examples are included directly in the Web application for making much more usable the application itself.

### Scenario 1: Integration of heterogeneous networks

Suppose that a cancer research laboratory is interested in investigating novel therapeutic approaches to lung cancer by studying the *TGF- β* signaling pathway and the role of the *SMADs* protein in the development of metastasis (epithelial-mesenchymal transition - EMT). By exploiting the functionalities of UNIPred-Web, the biologist can require the integration of the following three networks belonging to *Homo-sapiens* with respect to the Gene Ontology term GO:0001837 (i.e. EMT):

- STRING_v10 (id: 1980 – number of nodes: 3.632 – number of edges: 6.088 – category: STRING);
- Pathway.Wu-Stein-2010 (id: 1963 – number of nodes: 5299 – number of edges: 78.010 – category: Pathway);
- Physical_Interactions.IREF-BIOGRID (id: 1797 – number of nodes: 14.917 – number of edges: 155.470 – category: Physical Interactions).

The integration can be specified through the Integration and Prediction menu described in the paper.

The integration requires more or less three minutes for completing and produces an integrated network with 17.518 nodes and 503.926 edges. The user can wait that the process is completed and then click on the tab reporting the status of the computation. Alternatively, he can use the message that it is sent to the specified email address when the computation is completed. In this case, he can:

- click on the link that is received by mail (e.g. http://unipred.di.unimi.it/?load=2592), or
- copy and past the experiment identifier e.g. 2592 on the load button on the right top corner of the interface.

**Fig. 13:**
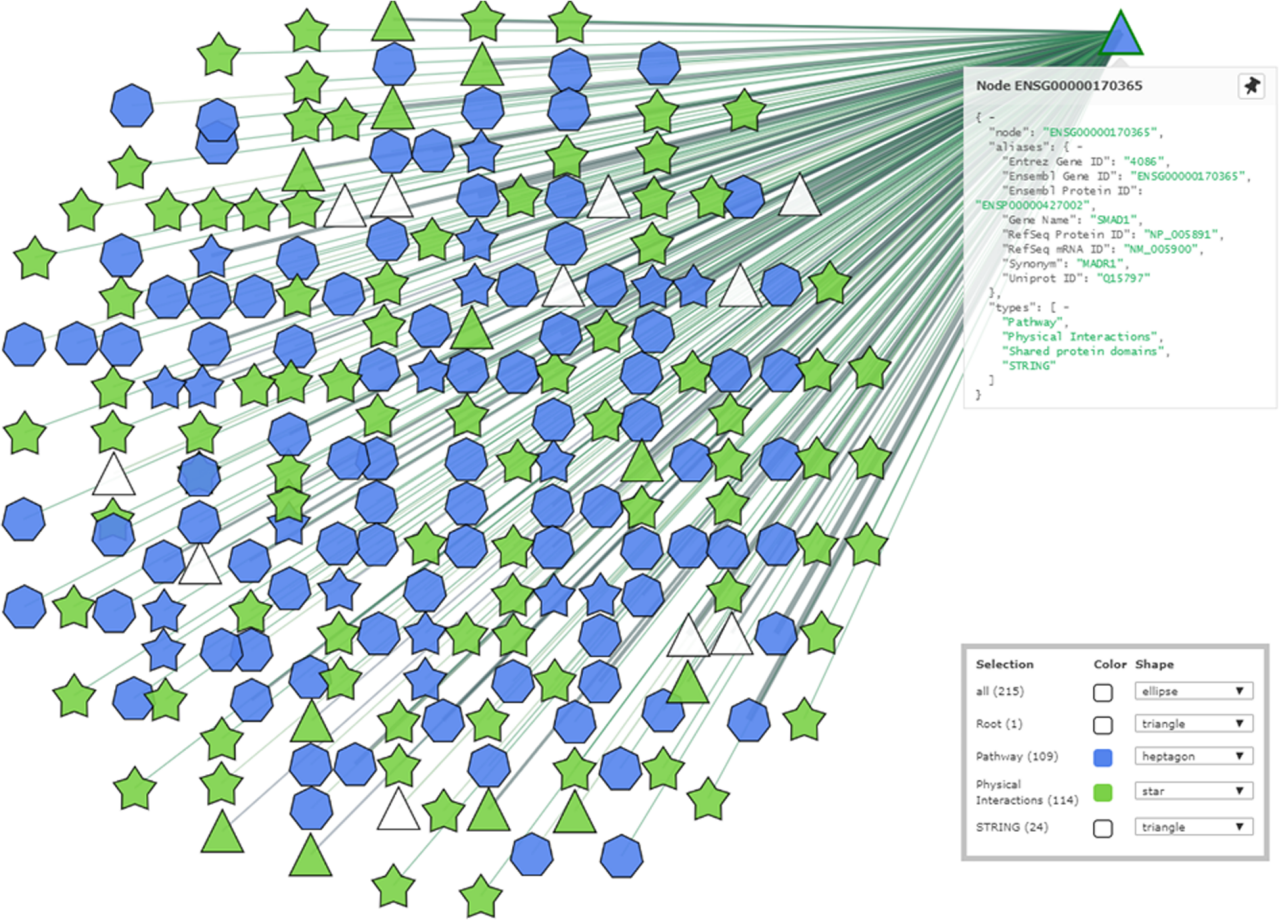
Exploration of the subnetwork for Scenario 1

Within the tab Integration-001-2592-View corresponding to the current integration, the user can look for the protein SMAD1. By using the search facility, the user points out that this biomolecule is internally represented through the Ensembl Gene IDENSG00000170365, and can select it as target protein of this experiment and proceeds with the visualization of the subnetwork centered on it. The target protein is highly connected with 214 proteins as shown in Fig. 13 and the user can use the different layout visualization options for improving the visual analysis of the integrated network. At the current stage the shape of the proteins that are directly connected with the target protein has been customized for pointing out the source from which they have been acquired (the used shapes are reported in the menu in the bottom right corner of Fig. 13).

From the integrated network, we can visualize a broader range of proteins connected to our target of interest and related to a specific protein function. Thus, we might unveil proteins related to our current study that are less investigated in literature and more difficult to detect from a simple literature search in such a systematic way. For example, we usually focus on proteins and pathways involved in the development, progression and metastasis of lung cancer and we are less up-to-date about new findings in different cancer types. For instance, by using UNIPred-Web integration module, we might decide to start studying a novel protein that could be involved in EMT but not yet extensively investigated in lungcancer.

**Fig. 14:**
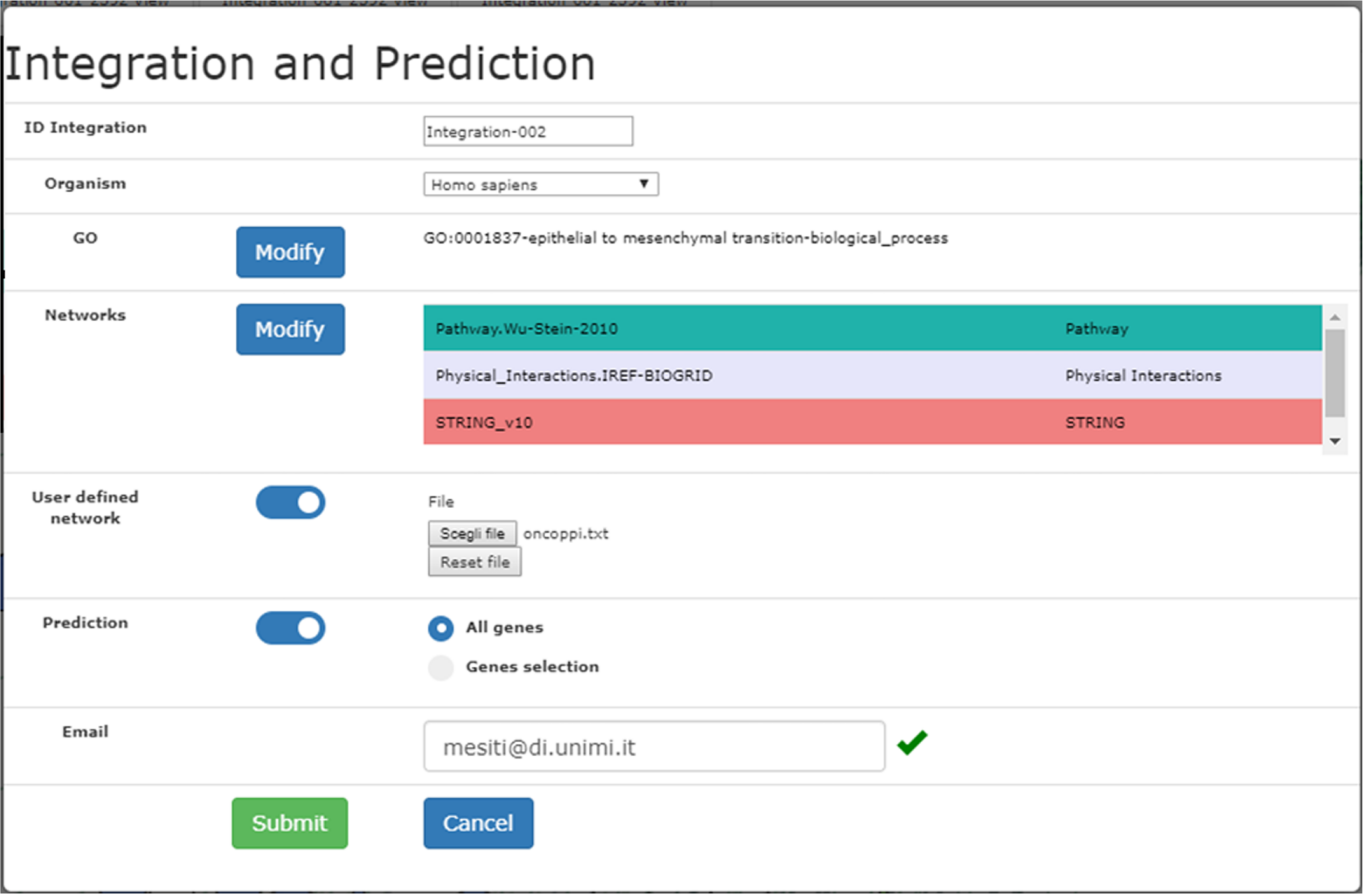
Specification of the Experiment for Scenario 2

### Scenario 2: Integration with user defined network and prediction

**Fig. 15:**
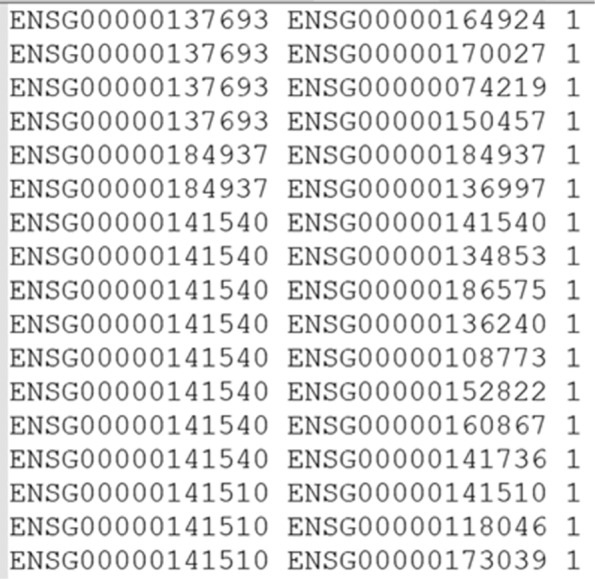
Excerpt of an user-defined network

Suppose now that the biologist wishes to identify “in-silico” a new protein that might be involved in the generation of metastasis of the lung cancer with the goal of studying the gene product later in vitro and/or in vivo. With this goal in mind, he decides to integrate in the UNIPred-Web database the OncoPPi network [38], which shows protein-protein interactions (PPIs) between cancer-associated proteins. He downloads the OncoPPi network file, available in the xgmml format (file ncomms14356-s4.zip) and the mapping file (file ncomms14356-s2.xlsx) between the gene-symbol (OncoPPi identifiers) and the ensemble-geneID (identifiers required by UniPred-Web). He downloads the OncoPPi network file, available in the xgmml format (file ncomms14356-s4.zip) and the mapping file (file ncomms14356-s2.xlsx) between the gene-symbol (OncoPPi identifiers) and the ensemble-geneID (identifiers required by UniPred-Web). This experimental scenario is shown in Fig. 14. Since the input network file required by UniPred-Web is the classical tab separated tuple (proteinID1 ∖tab proteinID2 ∖tab score), the OncoPPi network file is parsed by mapping the gene-symbol versus the ensemble-ID by using the gene-symbol as key. Finally, a network with 77 nodes and 397 interactions is obtained (no proteins/links are lost during the mapping phase). An excerpt of the considered network is reported in Fig. 15. The used scripts are available from [39].

**Fig. 16:**
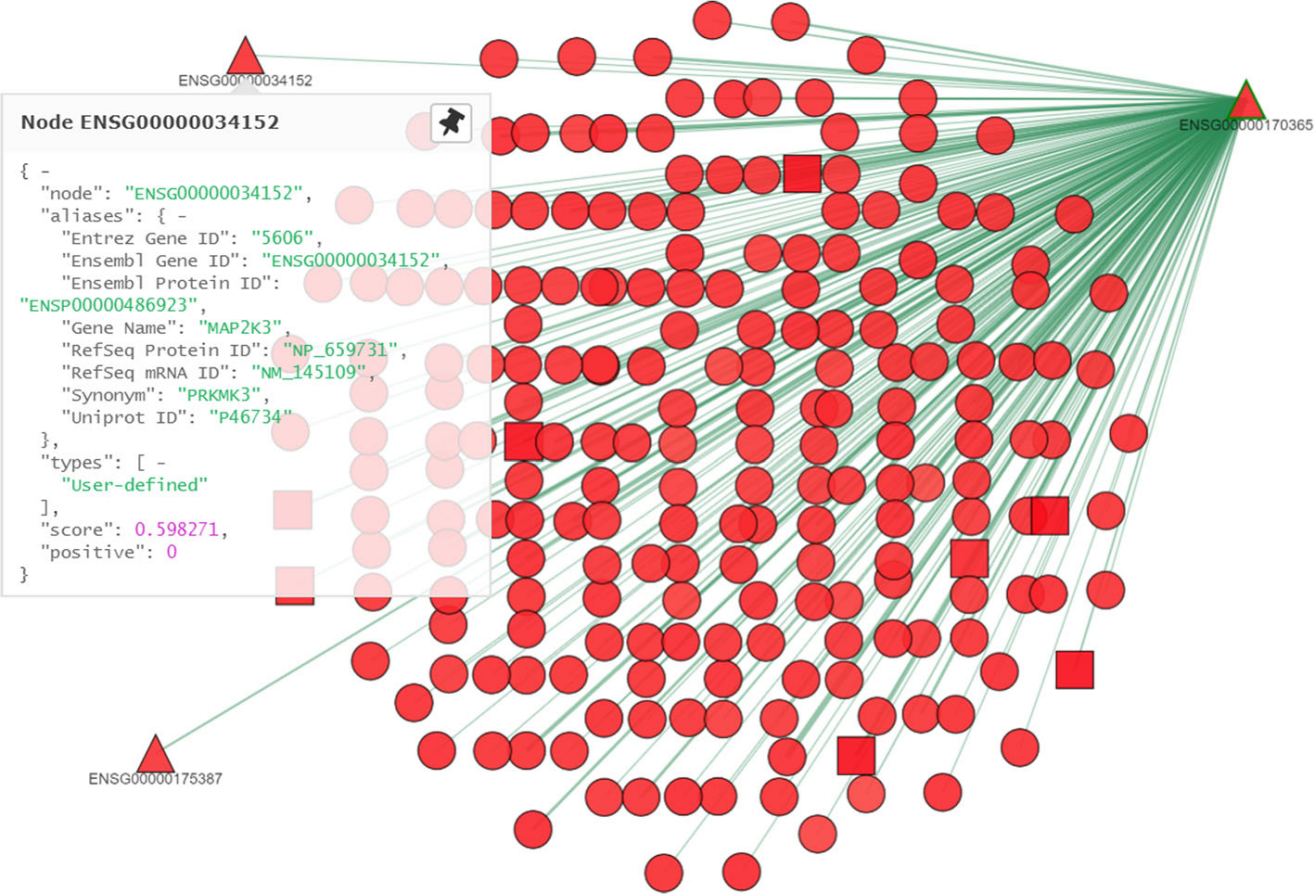
Exploration of the subnetwork for Scenario 2

By using the *Integration and Prediction* interface the previous three networks are selected and this user defined network is uploaded by activating the corresponding field. Moreover, also the prediction field is activated and the prediction all option selected. In this way, we can predict the class of unlabeled proteins using the COSNet algorithm. Also in this case, the integration takes few minutes for being completed and the experiment identifier 2594 is generated that can be used for conduction visual analysis. By inspecting the generated network from the target protein considered in Scenario 1 we can observe that two proteins are added to the integrated network (represented with a triangle) from the user defined network expressing a positive score with respect to the considered GO term as reported in Fig. 16.

We can also identify the first 8 proteins that are predicted as positive by COSNET: BAMBI, TGFB1, TGFB2, TGFBR1, BMP2, HMGA2, GSK3B and FRZB. It is worth noting that these proteins are annotated for the GO term EMT (GO:0001837) and some of them have been already described as involved in lung cancer, thus confirming UNIPred-Web as a valuable tool to visualize biomolecular networks and to perform protein function prediction. Moreover, by pressing the Predictions button (Current View option), the user can show a list of all COSNET predictions ordered by decreasing score, visualize, at the same time, whether the proteins already have an annotation (positive = 1, square shape in the network). Proteins without an annotation and with a high COSNET score may be possible candidates for further literature investigation followed by biological validation studies. For instance, the second ranked protein predicted by COSNET is ID4 (ENSG00000172201) which is related to EMT in lung cancer [40, 41]. This is another evidence that UNIPred-Web is able to correctly predict GO terms and that its integration in the daily research practice can facilitate the definition of new interesting targets for bio-medical research.

**Fig. 17:**
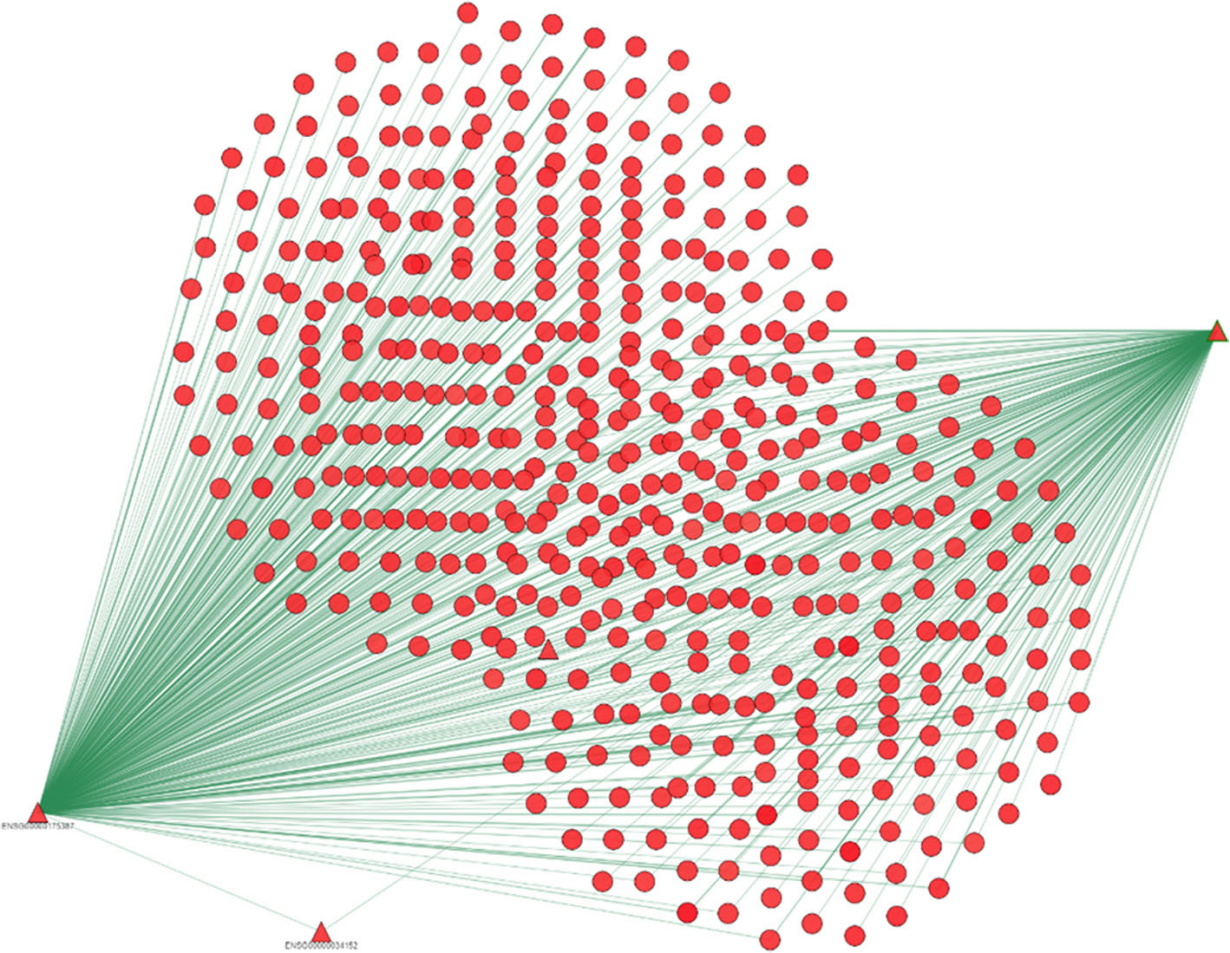
Navigation of neighbourhood proteins in Scenario 3

### Scenario 3: visual analysis and navigation

By using the facilities made available by the web application, we can inspect the characteristics of the network proteins and also the relationships among them. For example, in Fig. 16 the biologist can click on the protein ENSG00000175387 belonging to the user defined network and look at its characteristics. Specifically, he can see its aliases in other databanks, the types for which evidences have been acquired. Finally, the prediction score is reported, as well as the information that the protein is not a member of the training set (positive=0). The user can also expand the visualization along e.g. the protein ENSG00000175387 by clicking with the right mouse on the node and then selecting the option “one step from here”, thus adding to the network all the proteins in the neighborhood of the clicked node; by changing the position of the two proteins belonging to the user defined network the visualization in Fig. 17 can be obtained.

The visualization points out a relationship that only occurs in the user defined network between the proteins ENSG00000175387 and ENSG00000034152 and the target protein. Moreover, the target protein and the protein ENSG00000175387 are connected both directly and by means of several two steps paths. The three considered proteins SMAD1 (ENSG00000170365), SMAD2 (ENSG00000175387) and the protein MAP2K3 (ENSG00000034152) are part of two different pathways: TGF- *β* and Ras-MAPK. Since these three proteins are connected in our network, we can hypothesize that there is some sort of communication between the pathways. A literature search confirms that there is a non canonical crosstalk mechanism, associated with EMT in cancer cells, between TGF- *β* and Ras-MAPK [42]. This indicates that the option ”one step from here” can be used to highlight important intersections among pathways that require further investigations.

## Data Availability

**Project name:** UniPred-Web **Project home page:**http://unipred.di.unimi.it**Operating system:** Platform independent **Supported Browsers:** The Web application has been tested in several Operating systems (Windows 10, Mac OS Yosemite, Ubuntu 18 LTS) with the following browsers: FIREFOX (version 65.0 64bit), CHROME (version 73.0.3683.103 64 bit). On these browsers the application work perfectly without issues. The application works also well with Safari 10.1.22 on Mac OS Yosemite. We have also considered EDGE (version 44.17763.1.0) on Windows 10 and it works well with the exception of image download (which it is not supported). **Server-side Programming languages:** php (version 5), node.js (version 10.1.0), R-scripts (version 3.5) **Client-side Programming languages:** angular.js (version 1.2.16), cytoscape (version 3), jquery (version 2.2.4) **Database:** MariaDB 10 **License:** GNU GPL **Any restrictions to use by non-academics:** none The datasets are made available through the UniPred-Web application. Interested users can download the datasets directly from the application. For any concerns, the reader can make a request to the corresponding author.

## Abbreviations

AFP: Automated function prediction
CAFA: Critical assessment of functional annotation
COSNet: COst sensitive neural network
GO: Gene ontology
UNIPred: Unbalance-aware network integration and prediction
